# Caregiving Appraisals and Emotional Valence: Buffering Effect of Activity Participation

**DOI:** 10.1093/geroni/igab046.203

**Published:** 2021-12-17

**Authors:** Jeongeun Lee, Nicholas Cone

**Affiliations:** 1 Iowa State University, Ames, Iowa, United States; 2 Iowa State University, Iowa State University, Iowa, United States

## Abstract

Caregiving activities often lead to positive and negative appraisal for caregivers. Caregivers may limit social participation due to caregiving activities. Changes in level of activity participation could have profound consequences for caregiver’s valence. However, little is known about how activity participation could moderate the association between these caregiving appraisals and emotional valence. Data came from the National Study of Caregiving (Round 1 and 2), a nationally representative study of caregivers. Referencing Lawton’s two-factor model (1990), we examined both the level and changes in activity restriction interacting with positive and negative caregiving appraisals to predict both valence across two waves. Consistent with two factor models, findings revealed level and changes in activity restriction moderated the relationship between caregiving appraisal and outcomes for both valences. These findings highlight the role of activity restriction as a target to reduce negative valence and improve positive valence for caregivers.

